# An Unusual Case of Transthyretin Cardiac Amyloidosis Presenting as Heart Failure With Improved Ejection Fraction Without Left Ventricular Hypertrophy

**DOI:** 10.7759/cureus.83283

**Published:** 2025-05-01

**Authors:** Satoshi Kurisu, Hitoshi Fujiwara

**Affiliations:** 1 Department of Cardiology, National Hospital Organization (NHO) Hiroshima-Nishi Medical Center, Otake, JPN

**Keywords:** cardiac hypertrophy, carpal tunnel syndrome, scintigraphy, troponin, ventricular function

## Abstract

Heart failure with preserved ejection fraction (EF) and left ventricular (LV) hypertrophy (LVH) are typical phenotypes of transthyretin cardiac amyloidosis (ATTR-CA). Extracardiac manifestations, such as carpal tunnel syndrome, lumbar spinal stenosis, or biceps tendon rupture, frequently precede the development of overt cardiac involvement. We report a rare case of ATTR-CA presenting as heart failure with improved EF (HFimpEF) without LVH in an elderly patient, where the diagnosis was prompted by bilateral carpal tunnel syndrome, lumbar spinal canal stenosis, and persistently elevated troponin levels. An 88-year-old man presented with a two-week history of fatigue and anorexia. He had lumbar spinal canal stenosis and thenar muscle atrophy in both hands, suggestive of bilateral carpal tunnel syndrome. An electrocardiogram revealed right bundle branch block and left axis deviation, without low voltage or a pseudo-infarct pattern. An echocardiogram showed a reduced EF of 35% without LVH, with an LV end-diastolic diameter of 50 mm. Coronary angiography revealed no significant stenosis. He was diagnosed with non-ischemic heart failure with reduced EF. The presence of extracardiac red flags and elevated troponin levels raised suspicion for cardiac amyloidosis. 99m-Technetium pyrophosphate scintigraphy demonstrated Perugini grade 2 myocardial uptake (moderate uptake equal to rib uptake) on both planar and single photon emission tomographic images at the three-hour mark. No monoclonal protein was detected, and the free light chain ratio (kappa to lambda) was 1.54, within the normal range, leading to a non-invasive diagnosis of ATTR-CA. After 12 weeks of conventional heart failure therapy, follow-up echocardiography revealed an improved EF of 55% and a decrease in LV end-diastolic diameter to 35 mm, confirming the diagnosis of HFimpEF and indicating LV reverse remodeling and optimized volume status. In conclusion, this case illustrates that ATTR-CA can present atypically as HFimpEF, even in the absence of LVH or typical cardiac red flags. It highlights the importance of recognizing extracardiac signs and considering ATTR-CA in patients who do not exhibit typical features of LVH or traditional heart failure phenotypes.

## Introduction

Transthyretin cardiac amyloidosis (ATTR-CA) is an infiltrative cardiomyopathy caused by the extracellular deposition of transthyretin in the form of amyloid fibrils within the myocardium [1-3]. It includes two types: hereditary, due to transthyretin gene mutations, and wild type, related to age-associated misfolding mechanisms that remain poorly understood. Diagnosis is often delayed due to the non-specific nature of symptoms.

Although left ventricular (LV) hypertrophy (LVH) on echocardiography often serves as the initial diagnostic clue for ATTR-CA, extracardiac manifestations, such as carpal tunnel syndrome, lumbar spinal stenosis, or biceps tendon rupture, frequently precede the development of overt cardiac involvement [4,5]. Therefore, awareness of these extracardiac signs is crucial for enabling earlier detection and timely intervention.

While bone scintigraphy is an essential diagnostic tool for ATTR-CA [1-3], its high cost and limited availability restrict its use as a routine screening method. In this regard, recognizing extracardiac manifestations, although not mandatory, offers important diagnostic clues that can guide the selection of candidates for imaging and facilitate timely diagnosis.

Heart failure with preserved ejection fraction (EF) (HFpEF) and LVH have traditionally been considered the common phenotypes for ATTR-CA [1-3]. However, recent studies have shown a broad heart failure phenotypic spectrum, including heart failure with mildly reduced (HFmrEF) and reduced EF (HFrEF) [6,7]. Current guideline-directed medical therapy (GDMT) has been shown to reverse maladaptive cardiac remodeling and improve EF in patients with HFrEF [8,9]. Based on this, the term heart failure with improved EF (HFimpEF) has been defined as an increase in EF of at least 10 percentage points, from a baseline of less than 40% to 40% or greater [10]. HFimpEF has been associated with better prognosis compared with heart failure with persistently low EF [11]. This highlights the importance of achieving improved EF as a therapeutic goal, as it can lead to better long-term outcomes, including improved survival and quality of life. However, it remains unclear whether similar benefits of GDMT apply to patients with ATTR-CA, as these individuals have been systematically excluded from most major clinical trials.

Here, we report a rare case of ATTR-CA presenting as HFimpEF without LVH in an elderly patient, where the diagnosis was prompted by bilateral carpal tunnel syndrome and lumbar spinal canal stenosis, along with persistently elevated troponin levels.

## Case presentation

**Figure 1:**
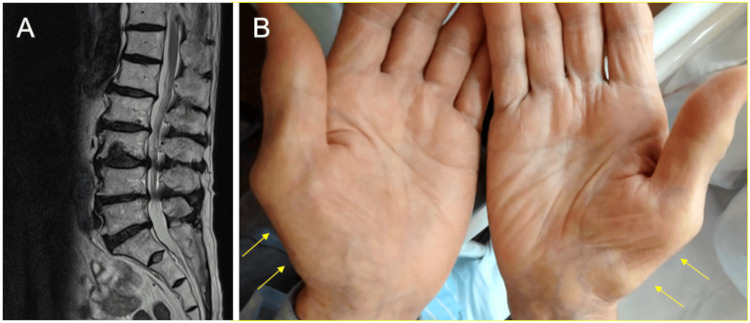
Extracardiac red flags for transthyretin cardiac amyloidosis (ATTR-CA) The patient had a history of lumbar spinal canal stenosis (A). On physical examination, he had thenar muscle atrophy in both hands (B, arrows), suggestive of bilateral carpal tunnel syndrome.

An 88-year-old man presented to our hospital with a two-week history of general fatigue and anorexia. No apparent triggers, such as overdrinking, overexertion, or infection, were identified. He had undergone a gastrectomy for gastric cancer five years earlier. The patient had a history of lumbar spinal canal stenosis (Figure 1A), but no family history of heart disease. On physical examination, his pulse rate was 84 bpm, with blood pressure of 94/66 mmHg, body weight of 54.2 kg, body mass index of 21.2 kg/m^2^, and oxygen saturation of 94%. Jugular vein distension was found in a sitting position, suggesting elevated right atrial pressure. He had pitting edema in both lower limbs and thenar muscle atrophy in both hands (Figure 1B, arrows), suggestive of bilateral carpal tunnel syndrome.

Laboratory studies revealed impaired hepatic function and increased troponin I level of 1,163 pg/mL (reference range: 0-26.2 pg/mL) (Table 1). The N-terminal pro-brain natriuretic peptide (NT-proBNP) level was elevated to 13,162 pg/mL (reference range: <126 pg/mL). He was admitted for further cardiac evaluation and treatment.

**Table 1:** Laboratory data

Variables	Initial presentation	17 days	7 weeks	12 weeks	Reference ranges
White blood cell count (/μL)	5.1 × 10^3^	－	6.4 × 10^3^	－	3.3-8.6 × 10^3^
Red blood cell count (/μL)	3.68 × 10^6^	－	3.77 × 10^6^	－	4.35-5.55 × 10^6^
Hemoglobin (g/dL)	11.6	－	11.8	－	13.7-16.8
Platelet count (/μL)	228 × 10^3^	－	233 × 10^3^	－	158-348 × 10^3^
Aspartate aminotransferase (U/L)	63	46	33	43	13-30
Alanine aminotransferase (U/L)	69	60	32	24	10-42
Lactate dehydrogenase (U/L)	391	242	245	241	124-222
Creatine kinase (U/L)	143	92	－	－	41-153
Creatine kinase-MB (U/L)	4.4	－	－	－	0-5
Total protein (g/dL)	6.3	－	－	－	6.6-8.1
Albumin (g/dL)	3.3	－	－	－	4.1-5.1
Blood urea nitrogen (mg/dL)	37.2	21.3	26.4	24.5	8-20
Creatinine (mg/dL)	1.02	0.91	1.00	1.07	0.65-1.07
Estimated gromerular filtration rate (mL/min/1.73 m^2^)	52.5	59.5	53.7	49.8	
C-reactive protein (mg/dL)	1.83	0.47	－	－	0-0.14
N-terminal pro-brain natriuretic peptide (pg/mL)	13,162	6,281	3,433	2,902	< 126
Troponin I (pg/mL)	1,163	63	87	－	0-26.2
Free light chain kappa (mg/L)	－	－	42.6	－	3.3-19.4
Free light chain lambda (mg/L)	－	－	27.7	－	5.7-26.3
Kappa-to-lambda ratio	－	－	1.54	－	0.26-1.65
Monoclonal protein	－	－	Not detected	－	

**Figure 2:**
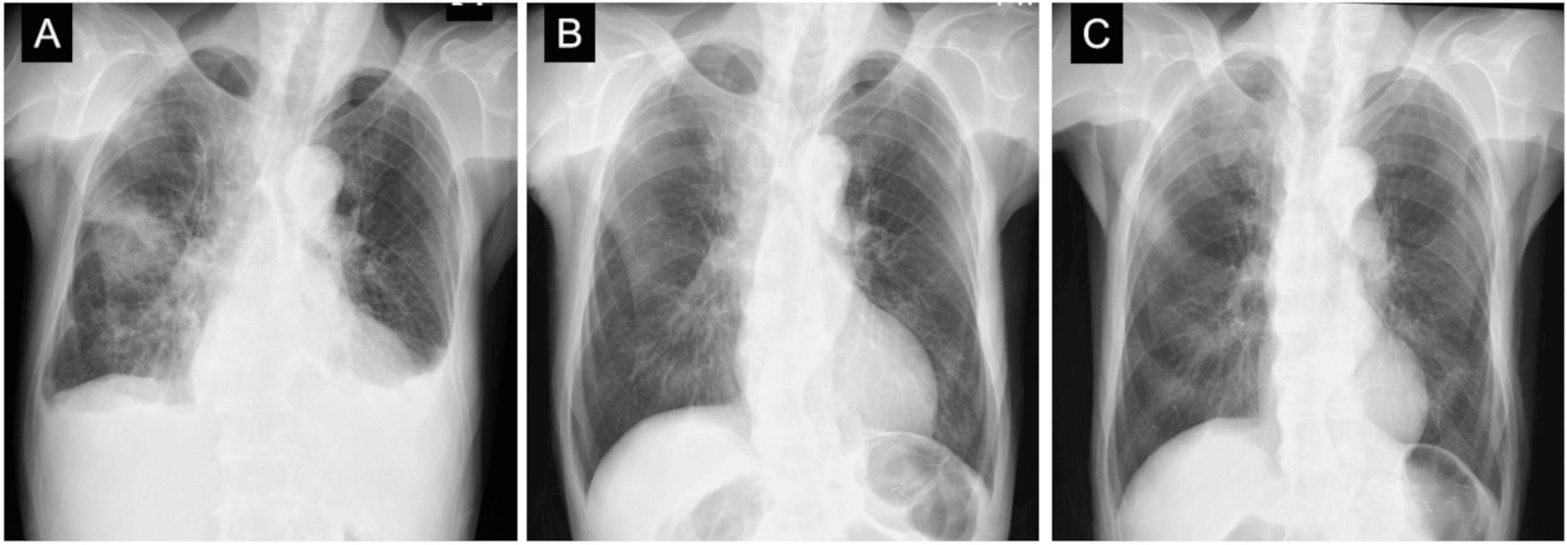
Chest radiographs A chest radiograph showed pulmonary congestion and pleural effusion at initial presentation (A). After two weeks of conventional heart failure therapy, the pleural effusion resolved, and the cardiothoracic ratio improved to 47% (B). After 12 weeks of conventional heart failure therapy, the cardiothoracic ratio further improved to 41% (C).

**Figure 3:**
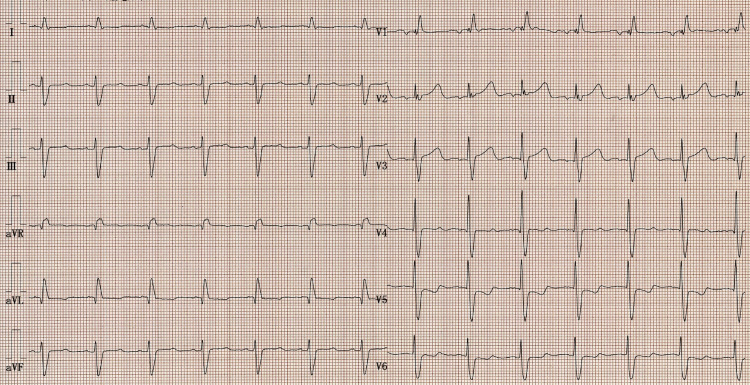
Patient's electrocardiogram (ECG) An ECG revealed the right bundle branch block and left axis deviation, without low voltage or a pseudo-infarct pattern.

A chest radiograph showed pulmonary congestion and pleural effusion (Figure 2A). An electrocardiogram (ECG) revealed right bundle branch block and left axis deviation, without low voltage or a pseudo-infarct pattern (Figure 3).

**Figure 4:**
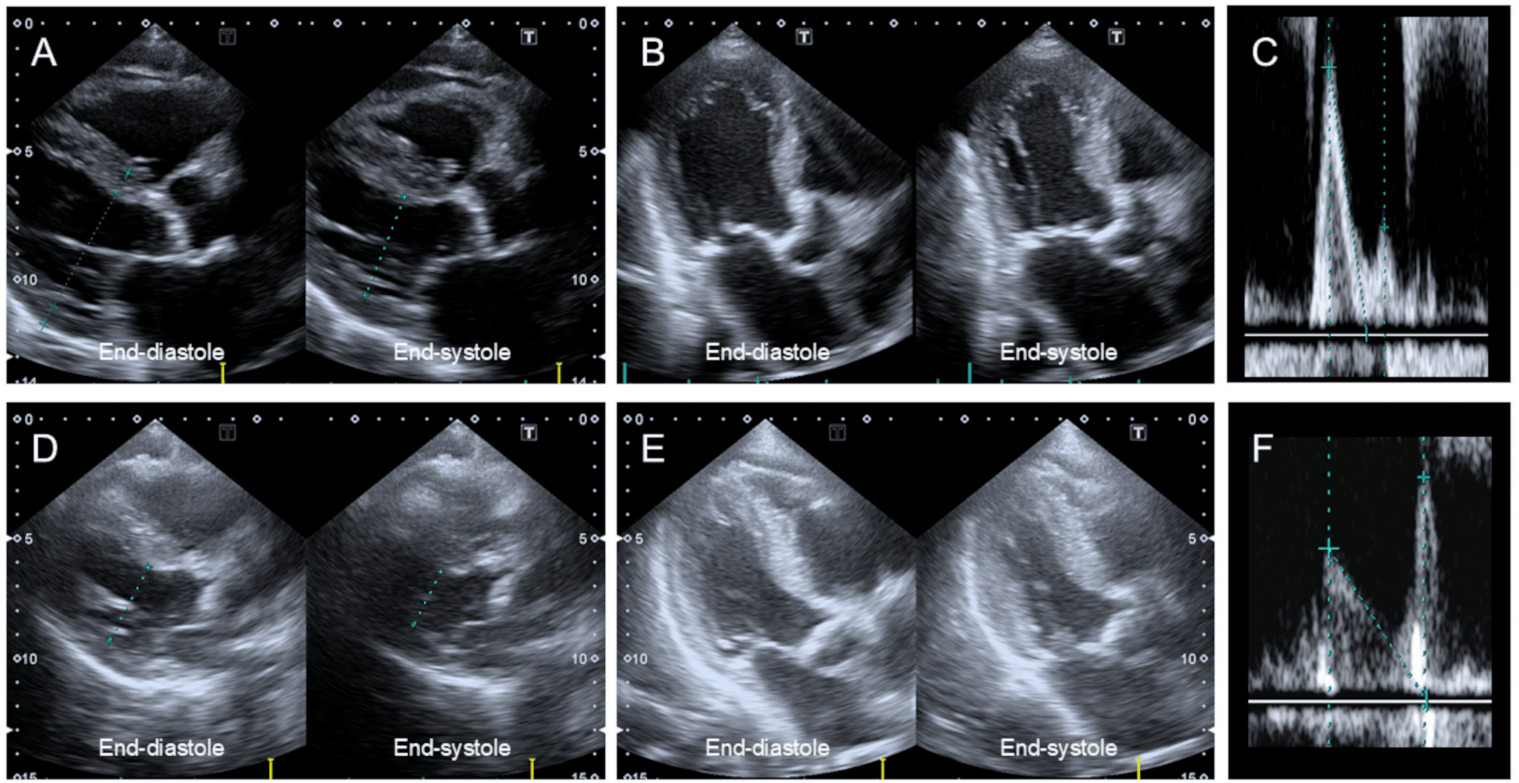
Transthoracic echocardiographic images A transthoracic echocardiography revealed a reduced EF of 35 % without LVH, with a LV end-diastolic diameter of 50 mm (A, B). Early to late diastolic mitral flow velocity ratio was 2.5, suggesting elevated left atrial pressure (C). After twelve weeks of conventional heart failure therapy, follow-up echocardiography revealed an improved EF of 55% (D, E), a decrease in LV end-diastolic diameter to 35 mm, and an early to late diastolic mitral flow velocity ratio of 0.7 (F), confirming the diagnosis of HFimpEF and indicating LV reverse remodeling and optimized volume status.

**Figure 5:**
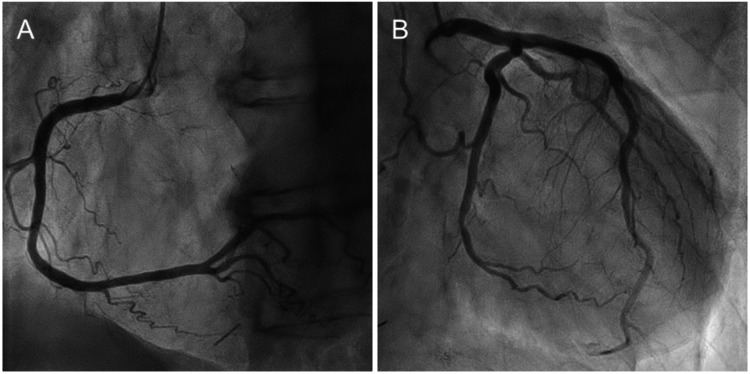
Coronary angiography Coronary angiography revealed no significant stenosis in the right (A) or left coronary arteries (B).

A transthoracic echocardiogram revealed a reduced EF of 35 % without LVH, with an LV end-diastolic diameter of 50 mm (Figures 4A-4B, Table 2). Interventricular septum thickness and posterior wall thickness were 10 mm and 8 mm, respectively. Mild aortic valve stenosis with a peak velocity of 1.7 m/sec and moderate mitral valve regurgitation were observed. The early-to-late diastolic mitral flow velocity ratio was 2.5, suggesting elevated left atrial pressure (Figure 4C). The inferior vena cava diameter at end expiration was 22 mm, with a collapsibility index of 36%, suggesting fluid overload. Coronary angiography revealed no significant stenosis in the right (Figure 5A) or left coronary arteries (Figure 5B).

**Table 2:** Transthoracic echocardiographic measurements LV, left ventricular

Variables	Initial presentation	12 weeks
LV end-diastolic dimension (mm)	50	35
LV end-systolic dimension (mm)	42	25
Interventricular septum thickness (mm)	10	10
LV posterior wall thickness (mm)	8	8
LV ejection fraction (%)	35	55
Early diastolic mitral flow velocity (cm/s)	113	66
Late diastolic mitral flow velocity (cm/s)	45	97
Early to late diastolic mitral flow velocity ratio	2.5	0.7
Severity of mitral regurgitation	Moderate	none
Severity of tricuspid regurgitation	Moderate	mild
LV outflow tract velocity time integral (cm)	9.5	11.8
Tricuspid regurgitation pressure gradient (mmHg)	32	23

**Figure 6:**
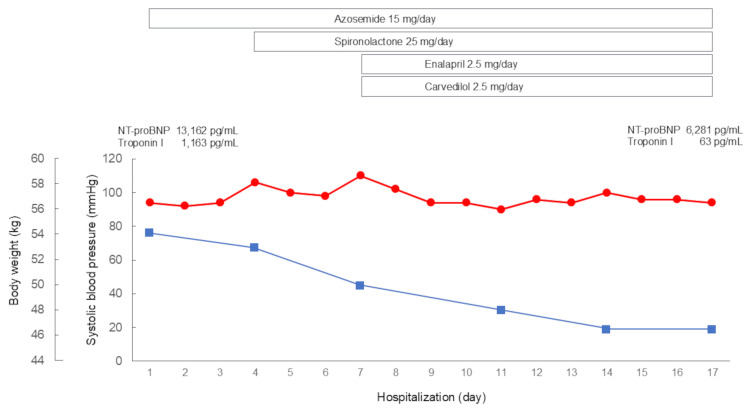
Clinical course during hospitalization Oral azosemide was started immediately after admission, and spironolactone, enalapril, and carvedilol were subsequently added. His symptoms gradually disappeared with decreasing body weight.

The patient was diagnosed with non-ischemic HFrEF [8]. Oral azosemide (15 mg/day) was started immediately after admission, and spironolactone (25 mg/day), enalapril (2.5 mg/day), and carvedilol (2.5 mg/day) were subsequently added (Figure 6). His symptoms gradually disappeared with decreasing body weight. After two weeks of conventional heart failure therapy, the pleural effusion resolved, and the cardiothoracic ratio improved to 47% (Figure 2B). NT-proBNP and troponin I levels improved after treatment but remained elevated. The patient was discharged on the 17th day of hospitalization.

**Figure 7:**
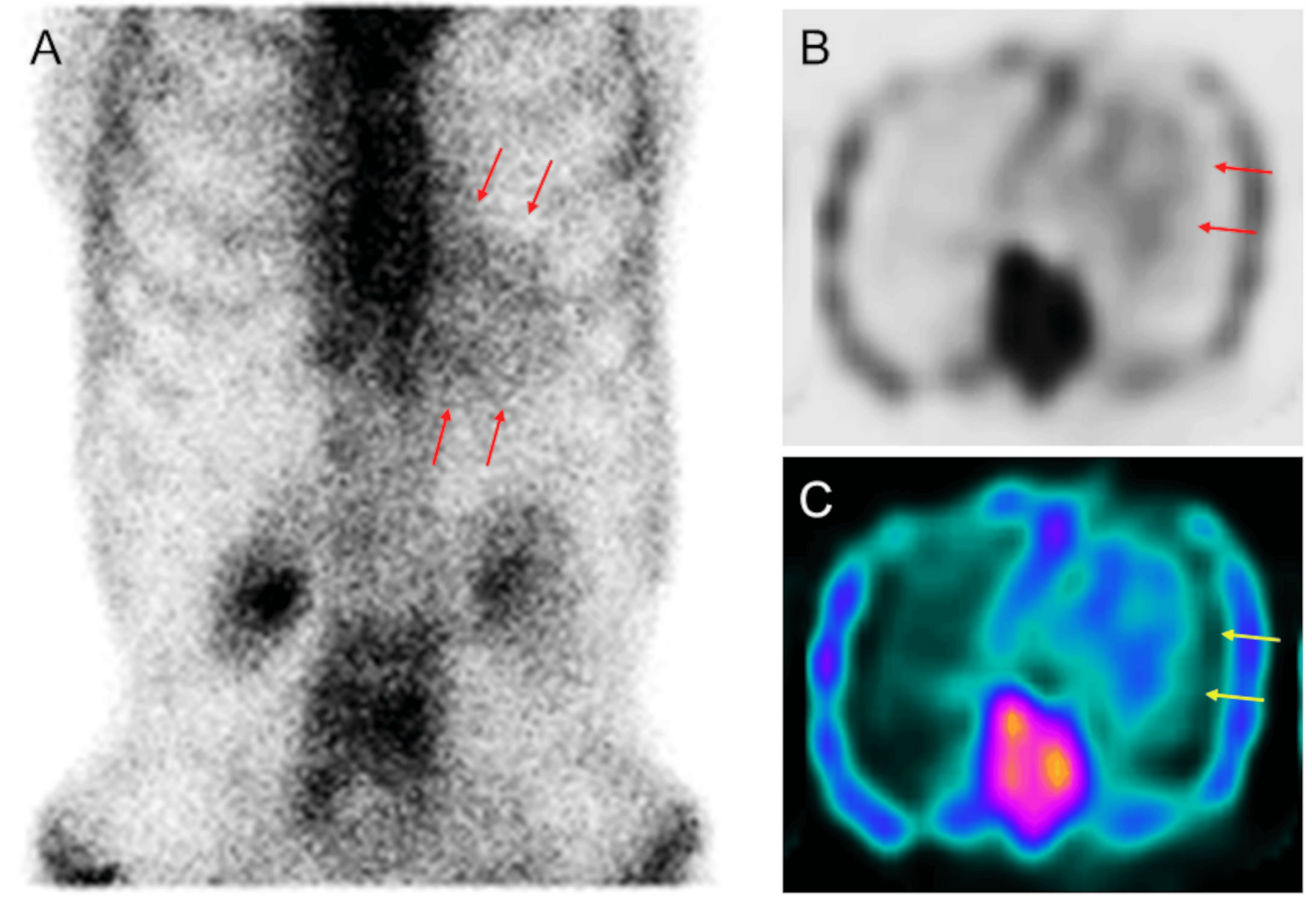
99mTc-PYP scintigraphy 99mTc-PYP scintigraphy demonstrated Perugini grade 2 myocardial uptake (moderate uptake equal to rib uptake), with an increased heart-to-contralateral ratio of 1.40, on both planar (A, arrows) and single photon emission tomographic images (B, C, arrows) at the three-hour mark.

Bilateral carpal tunnel syndrome and lumbar spinal canal stenosis, along with a persistent elevation in troponin levels, raised suspicion of cardiac amyloidosis, despite the absence of characteristic ECG and echocardiographic findings. The patient underwent diagnostic tests for cardiac amyloidosis. 99m-Technetium pyrophosphate (99mTc-PYP) scintigraphy demonstrated Perugini grade 2 myocardial uptake (moderate uptake equal to rib uptake), with an increased heart-to-contralateral ratio of 1.40, on both planar (Figure 7A, arrows) and single photon emission tomographic images (Figures 7B-7C, arrows) at the three-hour mark [12-16].

Serum and urine immunoglobulin electrophoresis revealed no abnormal bands, and the free light chain ratio (kappa-to-lambda ratio) was 1.54, which was within the normal range. At the family's request, neither an endomyocardial biopsy nor genetic testing for transthyretin mutations was performed. The patient was diagnosed with ATTR-CA non-invasively.

After 12 weeks of conventional heart failure therapy, the cardiothoracic ratio further improved to 41% (Figure 2C), while the ECG remained unchanged from the initial examination. Follow-up echocardiography revealed an improved EF of 55% (Figures 4D-4E, Table 2), a decrease in LV end-diastolic diameter to 35 mm, and an early to late diastolic mitral flow velocity ratio of 0.7 (Figure 4F, Table 2), confirming the diagnosis of HFimpEF and indicating LV reverse remodeling and optimized volume status. The patient remained stable during the four-month follow-up period.

## Discussion

In this report, we presented an unusual case of ATTR-CA presenting as HFimpEF without LVH. The diagnosis was prompted by extracardiac red flags, such as bilateral carpal tunnel syndrome and lumbar spinal canal stenosis, along with persistently elevated troponin levels.

HFpEF and LVH have traditionally been thought to be the common phenotypes for ATTR-CA [1-3]. A set of signs and symptoms, commonly known as red flags, has been proposed to aid in the early detection of ATTR-CA. Cardiac red flags include low QRS voltage or a pseudo-infarct pattern on ECG, along with increased LV wall thickness on echocardiography [1-3]. This case was considered atypical for ATTR-CA, as the patient initially presented with HFrEF in the absence of typical cardiac red flags. However, the presence of extracardiac red flags - including bilateral carpal tunnel syndrome and lumbar spinal canal stenosis - along with persistently elevated troponin levels [1-5], raised clinical suspicion and ultimately led to the diagnosis of ATTR-CA.

This case was notable for the development of HFimpEF, a relatively uncommon finding in the setting of ATTR-CA. Several studies have recently shown a broad heart failure phenotypic spectrum in cases of ATTR-CA [4,5]. Alonso et al. studied 213 patients with ATTR-CA confirmed by endomyocardial biopsy or 99mTc-PYP scintigraphy [6]. They demonstrated that 21.6% of patients were classified as HFrEF, 17.8% as HFmrHF, and 60.6% as HFpEF at the time of diagnosis. Martyn et al. also reported that, among 585 patients with ATTR-CA, HFrEF and HFmrEF were found in 28% and 17%, respectively [7]. These findings underscore the heterogeneity across LV function at the time of diagnosis. Neither of these studies specifically mentioned HFimpEF, as the focus was primarily on the classification of heart failure into the HFrEF, HFmrEF, and HFpEF categories.

In this case, the patient initially presented with HFrEF, with the underlying ATTR-CA remaining unrecognized. Currently, there are no established guidelines for the use of GDMT in the context of ATTR-CA and HFrEF, and its use remains controversial. A small case series of ATTR-CA patients with HFrEF treated with GDMT showed that 50% experienced improved EF and better functional capacity, while the other 50% remained decompensated and required hospice care [17]. Current GDMT for HFrEF includes four major classes of drugs: beta-blockers, mineralocorticoid receptor antagonists (MRAs), sodium-glucose cotransporter 2 (SGLT2) inhibitors, and angiotensin receptor-neprilysin inhibitors (ARNIs) [8,9]. However, in this case, we did not introduce SGLT2 inhibitor or ARNI due to the patient's frailty and low blood pressure, which made these therapies less suitable. Instead, conventional heart failure therapy with low-dose spironolactone, enalapril, and carvedilol led to an improvement in EF from 35% to 55%. In this case, we also decided not to introduce tafamidis treatment due to the patient's age, activities of daily living, and concerns over cost-effectiveness. Additionally, the need for further evaluation, especially myocardial biopsy, before considering the applicability of tafamidis in Japan, played a key role in this decision.

Recent cohort studies provide additional insight into GDMT in ATTR-CA. In a study by Ioannou et al., beta-blockers and angiotensin-converting enzyme inhibitors/angiotensin II receptor blockers were often discontinued, whereas MRAs were rarely stopped [18]. The propensity score-matched analysis showed that patients treated with beta-blockers (when EF was <40%) and MRAs had lower all-cause mortality [18]. Another large cohort study by Porcari et al. demonstrated that SGLT2 inhibitors were well tolerated and associated with reduced risks of all-cause mortality, cardiovascular mortality, and heart failure hospitalization in a propensity score-matched analysis [19]. Based on prior studies and our case, GDMT may be used cautiously in patients with ATTR-CA and HFrEF, as it may not be harmful and could offer benefits in select patients. However, treatment should be individualized, considering patient-specific factors such as frailty, blood pressure, and LV function. It is also important to note that, in our case, the etiology of HFrEF may not be entirely attributable to ATTR-CA alone. Other factors, such as age-related myocardial changes or unrecognized comorbidities, may have contributed to the reduction in EF. Further investigation is needed to better understand the complex interaction between ATTR-CA and other potential mechanisms contributing to HFrEF in this patient.

Another notable feature was the absence of LVH in the context of ATTR-CA. It is well recognized that amyloid deposition precedes the development of LVH, which typically becomes evident only in the later stages of the disease [20]. Advances in imaging modalities have made it possible to diagnose ATTR-CA in the pre-LVH phase through non-invasive approaches. In a pivotal study, Gillmore et al. demonstrated that the combination of grade 2 (moderate uptake equal to rib uptake) or grade 3 myocardial uptake (high uptake greater than rib uptake) on bone scintigraphy, along with the absence of monoclonal protein in serum or urine, provides a specificity and positive predictive value of 100% for the diagnosis of ATTR-CA [12]. Utilizing this diagnostic strategy, Devesa et al. evaluated 58 patients with HFpEF and no evidence of LVH, identifying ATTR-CA in three patients (5.2%). The median EF in these patients was 60%, and their median LV wall thickness was 11 mm [21]. In our case, the patient had a LV wall thickness of 10 mm and grade 2 myocardial uptake on 99mTc-PYP scintigraphy, indicating the pre-LVH phase of ATTR-CA. This early stage of the disease may have contributed to the improvement in EF following the initiation of conventional heart failure therapy.

This case report has limitations, as tissue diagnosis and genetic testing for ATTR-CA were not performed. A more comprehensive multimodal evaluation would have strengthened the diagnostic certainty and potentially provided further insights into the patient's condition. While the usefulness of the apical-sparing pattern in the diagnosis of ATTR-CA is well established, it was not available at our institution.

## Conclusions

In conclusion, this case illustrates that ATTR-CA can present atypically as HFimpEF, even in the absence of LVH or typical cardiac red flags. It highlights the importance of recognizing extracardiac signs and considering ATTR-CA in patients who do not exhibit typical features of LVH or traditional heart failure phenotypes. Early-stage diagnosis, even before the development of overt LVH, is therefore crucial for timely management and improved outcomes in patients with ATTR-CA. While early diagnosis can be challenging, it remains vital for optimizing patient care.
